# Sex-Related Disparities in the Incidence and Outcomes of Ischemic Stroke among Type 2 Diabetes Patients. A Matched-Pair Analysis Using the Spanish National Hospital Discharge Database for Years 2016–2018

**DOI:** 10.3390/ijerph18073659

**Published:** 2021-04-01

**Authors:** Ana López-de-Andrés, Rodrigo Jimenez-Garcia, Valentin Hernández-Barrera, Isabel Jiménez-Trujillo, José J. Zamorano-León, David Carabantes-Alarcon, Marta Lopez-Herranz, José M. de Miguel-Yanes, Javier de Miguel-Diez

**Affiliations:** 1 Department of Public Health & Maternal and Child Health, Faculty of Medicine, Universidad Complutense de Madrid, 28040 Madrid, Spain; anailo04@ucm.es (A.L.-d.-A.); josejzam@ucm.es (J.J.Z.-L.); dcaraban@ucm.es (D.C.-A.); 2 Preventive Medicine and Public Health Teaching and Research Unit, Health Sciences Faculty, Universidad Rey Juan Carlos, Alcorcón, 28040 Madrid, Spain; valentin.hernandez@urjc.es (V.H.-B.); isabel.jimenez@urjc.es (I.J.-T.); 3 Faculty of Nursing, Physiotherapy and Podology, Universidad Complutense de Madrid, 28040 Madrid, Spain; martal11@ucm.es; 4 Internal Medicine Department, Hospital General Universitario Gregorio Marañón, Universidad Complutense de Madrid, Instituto de Investigación Sanitaria Gregorio Marañón (IiSGM), 28009 Madrid, Spain; josemaria.demiguel@salud.madrid.org; 5 Respiratory Care Department, Hospital General Universitario Gregorio Marañón, Universidad Complutense de Madrid, Instituto de Investigación Sanitaria Gregorio Marañón (IiSGM), 28009 Madrid, Spain; javier.miguel@salud.madrid.org

**Keywords:** ischemic stroke, type 2 diabetes mellitus, sex differences, incidence, in-hospital mortality

## Abstract

Background: To analyze the incidence, use of therapeutic procedures, and in-hospital outcomes among patients suffering an ischemic stroke (IS) according to the presence of type 2 diabetes mellitus (T2DM) in Spain (2016–2018) and to assess the existence of sex differences. Methods: Matched-pair analysis using the Spanish National Hospital discharge. Results: IS was coded in 92,524 men and 79,731 women (29.53% with T2DM). The adjusted incidence of IS (IRR 2.02; 95% CI 1.99–2.04) was higher in T2DM than non-T2DM subjects, with higher IRRs in both sexes. Men with T2DM had a higher incidence of IS than T2DM women (IRR 1.54; 95% CI 1.51–1.57). After matching patients with T2DM, those with other comorbid conditions, however, significantly less frequently received endovascular thrombectomy and thrombolytic therapy. In-hospital mortality (IHM) was lower among T2DM men than matched non-T2DM men (8.23% vs. 8.71%; *p* < 0.001). Women with T2DM had a higher IHM rate than T2DM men (11.5% vs. 10.20%; *p* = 0.004). After adjusting for confounders, women with T2DM had a 12% higher mortality risk than T2DM men (OR 1.12; 95% CI 1.04–1.21). Conclusions: T2DM is associated with higher incidence of IS in both sexes. Men with T2DM have a higher incidence rates of IS than T2DM women. Women with T2DM have a higher risk of dying in the hospital.

## 1. Introduction

Diabetes is a well-known, independent, and strong risk factor for neurovascular disease [1], particularly ischemic stroke. Patients with diabetes have been found to have higher risk of ischemic stroke than no diabetic subjects [2]. Additionally, diabetes is frequent in patients with stroke, with a prevalence of approximately 20–33% [3,4,5] and has been associated with a higher incidence of long-term vascular complications, a worse functional outcome, and more in and out hospital mortality, when compared with patients without diabetes [6].

Previous investigations have reported that a low-grade immune activation represents an important risk factor not only for the development of type 2 diabetes mellitus (T2DM) but also for several vascular macrovascular complications of diabetes such as myocardial infarction and stroke [7,8]. Involvement of inflammatory markers such as IL−6 plasma levels and resistin in diabetic subjects confirmed the pathogenetic issue of the “adipovascular” axis that may contribute to cardiovascular risk in patients with T2DM. Elevated serum resistin concentration appears to be an independent risk factor for ischemic stroke. In contrast with resistin, adiponectin is known to enhance insulin sensitivity and also exhibits antiinflammatory and atheroprotective actions in various tissues [7,8].

Sex differences may be related to the incidence and outcomes of ischemic stroke among persons with diabetes [9,10,11]. Peters et al. [9] concluded that diabetes had a greater impact in the risk of stroke among women than men (27% excess risk), independently of other stroke risk factors. Population-based studies in Spain showed that women with type 2 diabetes (T2DM) had poorer hospital outcomes than diabetic men and diabetes increased the risk of in-hospital mortality (IHM) only in women (OR 1.07; 95% CI 1.05–1.11) [12,13]. Nevertheless, several studies have reported contradictory results regarding sex difference in the risk of stroke associated with diabetes [14,15]. A huge study conducted in the UK, with a sample of almost two million subjects, found no sex differences in the association between diabetes and stroke subtypes [14].

The expected increase in the prevalence of T2DM worldwide in future decades, and the association of diabetes and ischemic stroke may result in a significant burden on medical costs, therefore making it necessary to investigate this topic [16]. The objectives of this investigation were to assess the differences, according to the presence of T2DM and sex, in the incidence of patients admitted with a primary diagnosis of ischemic stroke and to compare their clinical characteristics and in-hospital outcomes. We also tried to assess which variables were independently associated with a higher mortality after hospitalization by ischemic stroke among men and women with T2DM.

## 2. Materials and Methods

### 2.1. Study Design and Data Source

This was an epidemiological retrospective observational study. The database used was the Spanish National Hospital Discharge Database (SNHDD). This database is managed by the Spanish Ministry of Health and collects over 95% of all hospital (public and private) discharges in Spain. The SNHDD uses the International Classification of Disease version 10 (ICD-10) for coding. The variables and characteristics of the SNHDD can be found online [17].

### 2.2. Study Population

We analyzed data from all subjects aged ≥35 years hospitalized and recorded in the SNHDD from 1 January 2016 to 31 December 2018.

Our study population included subjects discharged with a primary diagnosis of ischemic stroke identified with the ICD-10 codes shown in Supplementary Table S1 [17].

The population was stratified according to sex and the presence or not of a T2DM diagnosis code (E11.x) in any diagnosis position (2–20). If a code for T1DM (E10.x) was found in any diagnosis field the patient was excluded. 

### 2.3. Study Variables 

Our main outcome variables of interest included the incidence of ischemic stroke, IHM, and length of hospital stay (LOHS). We also analyzed the use of endovascular thrombectomy and thrombolytic therapy during the hospitalization.

To calculate the incidence of ischemic stroke hospitalizations we used as denominator the number of subjects with and without T2DM. These numbers were obtained using the age and sex groups prevalence estimations of T2DM provided by the Spanish National Health Survey 2017 multiplied by the population in each age and sex stratum according to the Spanish National Statistics Institute [18]. 

Independent variables included sex, age, comorbidities, and procedures conducted during the hospital admission. To quantify comorbidity, the Charlson Comorbidity Index (CCI) was calculated for each patient with the algorisms described by Sundararajan et al. [19]. 

We also specifically described and compared the prevalence among patients with and without T2DM of cardiovascular risk factors (obesity, hypertension, or lipid metabolism disorders), chronic diseases present at admission (renal disease, acute myocardial infarction, atrial fibrillation, congestive heart failure, dementia, anemia, peripheral vascular disease, alcohol abuse, or depression), and therapeutic procedure (mechanical ventilation, endovascular thrombectomy, or thrombolytic therapy). The ICD10 codes used appear in Supplementary Table S1 and [19].

### 2.4. Matching Method

We matched, within the SNHDD database, each man with T2DM with a non-diabetic man of the same age, type of ischemic stroke (up to second ICD 10 codes digits), and year of hospitalization. The same process was done for each woman. In addition, to assess sex differences, pair-matching was done for each T2DM women with a T2DM men using the variables described previously.

### 2.5. Statistical Analysis

Statistical analysis was conducted separately for women and men. 

Descriptive statistics for continuous variables were reported as means with standard deviation or medians with interquartile range and with absolute frequency and percentage for categorical variables.

To estimate the differences in the incidence between study groups the statistical method used was Poisson regression. We constructed models adjusted by age and sex or by only age depending in the groups being compared. 

Student’s *t*-test or Mann–Whitney tests were used to compare means and medians, respectively. Prevalence and proportions were compared with Chi-square tests.

Multivariable logistic regression was constructed, using IHM as the dependent variable, to identify those variables independently associated with dying during the hospital admission after ischemic stroke. Separate models for men, women, and both sexes, according to the presence of T2DM, were constructed as described before [18].

Stata version 14 (Stata, College Station, TX, USA) was the statistical software for matching and descriptive and bivariate and multivariable analytical statistics. Two-sided *p*-value of <0.05 was the cut-point used for significance. 

### 2.6. Ethical Aspects

The SHDD database can be requested by any researcher at the Spanish Ministry of Health using the online questionnaire [20]. According to Spanish law, the use of anonymized databases provided by official bodies does not require authorization from an ethics committee. This is so because the Ministry of Health only transfers the data if all ethical requirements are guaranteed.

## 3. Results

The number of hospital discharges in Spain from 2016 to 2018 for patients aged 35 years or over with a primary diagnosis of ischemic stroke was 172,255 (29.53% with T2DM). Men represented 53.71% (*n* = 92,524) and women 46.29% (*n* = 79,731) of this total. The overall prevalence of T2DM was higher among men than women (30.95% vs. 27.87%; *p* < 0.001).

### 3.1. Incidence of Ischemic Stroke According to T2DM

As can be seen in Table 1, the total incidence of ischemic stroke was higher (*p* < 0.001) among the T2DM population (111.61 per 100,000 persons with T2DM) than among those without T2DM (27.93 per 100,000 persons without T2DM) resulting in an adjusted IRR of 2.02 (95% CI 1.99–2.04). 

According to sex we found that among men with T2DM the ischemic stroke adjusted incidence was around twice (124.68 vs. 30.83; IRR 2.19 95% CI 2.16–2.22) that of non-T2DM men. Among women with T2DM, the incidence of ischemic stroke was also significantly higher than among non-T2DM women (adjusted IRR 1.77; 95% CI 1.75–1.80).

Men with T2DM had higher adjusted incidence of ischemic stroke than T2DM women (IRR 1.54; 95% CI 1.51–1.57). Among men without T2DM the adjusted incidence was also higher than in non-T2DM women (IRR 1.51; 95% CI 1.49–1.53).

### 3.2. Clinical Characteristics and Hospital Outcomes for Men and Women with Ischemic Stroke According to T2DM

The clinical characteristics, therapeutic procedures, and hospital outcomes before and after matching by age and ischemic stroke type for men and patients with ischemic stroke are shown in Table 2.

Men with T2DM matched by age and ischemic stroke type had more comorbid conditions than non-diabetic men. The prevalence of obesity, hypertension, lipid metabolism disorders, renal disease, congestive heart failure, peripheral vascular disease, acute myocardial infarction, dementia, anemia, and depression were significantly higher. However, the prevalence of atrial fibrillation was higher among non-diabetic men (22.33% vs. 21.06%; *p* < 0.001). Non-T2DM men had more frequently received mechanical ventilation (3.02% vs. 2.21%; *p* < 0.001), endovascular thrombectomy (4.46% vs. 2.91%; *p* = 0.034), and thrombolytic therapy (7.29% vs. 5.45%; *p* < 0.001). IHM was significantly higher in non-T2DM men than in men with T2DM (8.71% vs. 8.23%; *p* < 0.001). 

When we compared women with and without T2DM who suffered ischemic stroke before and after matching we obtained the results shown in Table 3. As described for men, women with T2DM had significantly higher prevalence of most clinical conditions than matched non-diabetic women. Unlike in men, the prevalence of atrial fibrillation was higher among women with T2DM (14.19% vs. 8.39%; *p* < 0.001). 

Prevalence of alcohol abuse was higher in non-diabetic women than in women with T2DM (1.18% vs. 0.81%; *p* < 0.001).

Regarding procedures and hospital outcomes we found that women with T2DM had less frequently had a code for mechanical ventilation (2.12% vs. 1.78%; *p* = 0.011), endovascular thrombectomy (5.02% vs. 2.91%; *p* < 0.001), or thrombolytic therapy (7.35% vs. 5.67%; *p* < 0.001). 

LOHS was significantly higher in T2DM women. The IHM (13.28% for T2DM women and 12.91% for non-diabetic women) showed no significant difference after matching.

### 3.3. Clinical Characteristics and Hospital Outcomes for Diabetic Patients with Ischemic Stroke According to Sex

As can be seen in Table 4, after matching, diabetic women who suffered ischemic stroke had a higher mean CCI than diabetic men (0.72 vs. 0.67; *p* < 0.001). Specifically, females had higher prevalence of most conditions included in the CCI, except for renal disease (13.05% vs. 14.76%; *p* < 0.001), peripheral vascular disease (3.83% vs. 8.86%; *p* < 0.001), and acute myocardial infarction (3.99% vs. 7.11%; *p* < 0.001). Furthermore, men had a higher prevalence of alcohol abuse than females (7.74% vs. 0.95%; *p* < 0.001).

Use of mechanical ventilation, endovascular thrombectomy, and thrombolytic therapy showed no significant difference among men and women. The overall median LOHS was 7 days in men and women and IHM rate was 11.15% for females and 10.20% for males (*p* = 0.004).

### 3.4. Multivariable Analysis of Variables Associated with IHM among Men and Women with T2DM

The results of the multivariable logistic regression analysis among diabetic patients with ischemic stroke are shown in Table 5.

For men and women, the risk of dying in the hospital increased with age, renal disease, atrial fibrillation, congestive heart failure, acute myocardial infarction, dementia, and the need of mechanical ventilation during the hospitalization. 

Obesity and undergoing thrombolytic therapy reduced the IHM in both sexes. 

We found that women with T2DM have a significantly higher probability of dying in the hospital than T2DM men (OR 1.12, 95% CI 1.04–1.21).

Finally, using the entire database including men and women with ischemic stroke and after multivariable adjustment (Table S2), we found no differences in the IHM rate according to diabetes status for men (OR 1.03; 95% CI 0.97–1.1) and women (OR 1.06; 95% CI 0.99–1.13).

## 4. Discussion

This nationwide population-based observational study showed that men and women with T2DM had higher incidence rates of ischemic stroke than men and women without T2DM in all age groups analyzed. After pair-matching according to age, ischemic stroke code, and year of hospitalization, use of mechanical ventilation, endovascular thrombectomy, and thrombolytic therapy was lower in T2DM patients. IHM was significantly lower in men with T2DM than in non-diabetic men. Proceeding with thrombolytic therapy appeared to be associated with a lower IHM among T2DM patients. In the fully adjusted model, women with T2DM had a 12% higher adjusted risk of dying in the hospital after ischemic stroke than men with T2DM. 

According to our database, patients with diabetes had higher incidences of ischemic stroke than those without diabetes, irrespective of their sex. This finding has been previously reported [2,11,12,13]. Furthermore, T2DM men had higher incidence rates of ischemic stroke than T2DM women. These trends are in accordance with what had been previously described in diabetic population [10,21]. Despite the American Heart Association/American Stroke Association having summarized the particularities of ischemic stroke in women, in part due to the increasing rates stroke mortality in women [22], virtually every study shows persistently higher ischemic stroke incidence rates in men. What is striking is that these higher rates have been claimed to be incompletely explained by established risk factors [23]. As has been previously reported by other authors those suffering diabetes have a higher number of cardiovascular risk factors and concomitant chronic diseases [24,25]. Therefore, the IHM and long-term mortality rate in ischemic stroke patients with diabetes are 1.13-fold and 1.52-fold higher than in those without diabetes [26]. In addition, as expected, and consistent with findings reported before, older age, renal disease, atrial fibrillation, congestive heart failure, acute myocardial infarction, and dementia are risk factors for IHM [12,13,16,26,27].

In recent years, several studies have assessed the role of inflammation and the underlying cellular and molecular mechanisms that contribute to atherogenesis [7,8,28,29]. In addition to the effect of specific inflammation markers mentioned previously, it has been found that both aortic stiffness and wave reflection indexes are related to the degree of systemic inflammation in stroke subjects [7,8,28,29]. Stroke subjects with acute ischemic stroke and metabolic syndrome show a higher degree of immuno-inflammatory and arterial stiffness indices possibly due to metabolic background of these types of patients that trigger a more intense immune–inflammatory activation irrespective of stroke subtype [28,29].

The results of the present study indicate that during admission for ischemic stroke, men and women with T2DM undergo mechanical ventilation, endovascular thrombectomy, and thrombolytic therapy less frequently than matched non-T2DM men and women. Several studies have indicated that diabetes independently predicts worse functional outcomes after endovascular stroke therapy [30,31]. Recently, Panni et al. found that diabetes was an independent predictor of 90-day mortality after this procedure (OR, 3.23; 95% CI 1.34–7.8; *p* = 0.009) [32]. However, in the present study we did not find differences in the IHM rate in patients with T2DM according to endovascular thrombectomy, making it necessary to investigate the reasons for these differences. Mechanical ventilation is a well-known risk factor of mortality after ischemic stroke in patients with T2DM, as described in the literature [33].

Men with diabetes admitted with ischemic stroke have a lower IHM rate compared with men without diabetes. The current results reinforce those previously found in Spain, where for the period between 2003 and 2012, the IHM after an ischemic stroke was 9.68% for men with T2DM and 10.66% among those who did not suffer from T2DM (*p* < 0.001). [12]. Furthermore, Lau et al. concluded that in patients who had suffered an ischemic stroke, suffering from diabetes implied worse clinical results but not higher mortality within the hospital. In addition, these authors identified the increase in HbA1c levels as a negative prognostic factor after an ischemic stroke [16].

During admission for ischemic stroke, we found that being female and having diabetes was associated with a higher IHM, and the higher mortality risk among women with diabetes remained after the multivariable regression model analysis. The results of two large prospective studies, the UK Prospective Diabetes Study and the Monitoring of Trends and Determinants in Cardiovascular Disease (MONICA) study, both showed that women with diabetes had more than twice the risk of dying from stroke than men with diabetes [34,35]. In Spain, results of a prospective single-center stroke registry including 561 diabetic stroke patients, showed that there was higher in-hospital mortality among women than men (14.9% vs. 8.3%; *p* = 0.02) [36]. Studies from other countries reached similar conclusions [37]. Recently, Wang et al. conducted a meta-analysis including 15 studies with 2,292,387 subjects suffering stroke, assessing the sex differences in the mortality rate associated with diabetes. The pooled multiple-adjusted risk rate ratio (RRR) showed that women with diabetes had an 8% higher risk of mortality than diabetic men (RRR 1.08, 95% CI 1.01–1.15 *p* < 0.001) [38].

From a pathophysiological point of view sex-differences in cerebrovascular complication among T2DM subjects include biological factors like genetic predisposition, sex hormones, and neuro–humoral pathways, as well as psychosocial, behavioral, and environmental factors [38,39,40]. It should be stressed that biological factors are often influenced by psychosocial factors and it is likely that these interactions ultimately determine the differences in pathophysiology of vascular complications between women and men [39].

Experimental studies point to different responses to cerebral ischemia in female and male cells [41]. Furthermore, animals with an XX chromosome complement had larger infarcts and neurologic deficit scores, and greater immune-cell infiltration and activation, compared to animals with an XY chromosome complement [42]. 

Sex hormone studies have reported that T2DM is characterized by reduced levels of ovarian hormones alongside increased levels of testosterone and this change in hormone balance has been reported to be associated with cardiovascular diseases [38,39,40]. Lower estrogen levels in postmenopausal women reduce the anti-inflammatory and neuroprotective effects of the hormone. Therefore, the high-inflammatory environment produced by diabetes, combined with lower neuroprotection from estrogens, may aggravate brain damage, and consequently increase morbidity and mortality outcomes in women with diabetes [10,39].

Sex differences in coagulation and fibrinolysis, rate of progression of atherosclerosis and, endothelial function in individuals with diabetes have also been associated to different outcomes after stroke [9,10,39].

Differences in body anthropometry, patterns of storage of adipose tissue, and lipid metabolism may be of particular importance in explaining the sex differences in the diabetes-associated risk of vascular disease [39,43,44,45]. There is compelling evidence that obesity and its associated metabolic dysfunction suppresses women’s protective effect of sex-hormones on cardiovascular disease [39,43,44,45]

Deterioration in cardiovascular risk factor levels among those with T2DM is greater in women than in men; therefore, women with diabetes are disadvantaged compared with men, even before their diagnosis [45,46]. 

Regarding environmental factors, differences in treatment and management may explain a large component of the excess risk associated with diabetes in women [47,48]. In Spain women with diabetes are less likely than men with diabetes to meet all recommended care requirements and might be less likely to achieve target values for treated cardiovascular risk factors [47]. Interestingly, it has been recently demonstrated that poor control of blood sugar has a greater effect on the risk of stroke in the female gender [49].

Finally, worse outcomes among diabetic women may result in different clinical presentation or worse access to adequate therapies or diagnostic procedures when compared with men [48]. The multifactorial causes for excess risk among T2DM women requires further investigations.

Interestingly, obesity reduced the IHM for ischemic stroke in our investigation. The presence of an “obesity survival paradox” in patients with diabetes following an ischemic stroke remains controversial. Several studies showed discordant results by reporting positive [50,51], inverse [52], and U-shaped associations [53] between obesity and diabetes-related complications. Recently, a prospective cohort study from the European Prospective Investigation into Cancer and Nutrition (EPIC)-Postdam concluded that there was no apparent association of pre-diagnosis BMI and BMI change with the incidence of stroke and myocardial infarction (HR 1.04; 95% CI 0.62, 1.74) [54].

The strength of our investigation includes the large sample size (172,255 episodes of ischemic stroke, 29.53% with T2DM), the widespread coverage of the population of an entire country (>95% of all hospital admissions), the standardized methodology, and the good reliability of ischemic stroke coding in the SNHDD [55]. Yet, we should point out several limitations. Our data source is an administrative database that is supported by the information that physicians recorded in the discharge report, which also depends on manual coding on behalf of the administrative staff. Unfortunately, the SNHDD only includes up to 20 diagnoses and 20 procedures for each patient hospitalized using ICD-10 for coding. This coding system provides information on the artery affected (specific pre-cerebral or cerebral arteries) and whether the occlusion was due to embolism or thrombosis but not any other clinical data regarding the stroke characteristics or consequences. Therefore, no information is available in the SNHDD to evaluate the distribution of Trial of Org 10172 in Acute Stroke Treatment (TOAST) subtype of stroke, nor to quantify stroke severity using the National Institute of Health Stroke Scale/Score (NIHSS) or the degree of disability at discharge in our epidemiological study. However, administrative databases using the ICD coding system have been previously used to investigate stroke in Spain and other countries [11,12,13,14,26,37]. 

Despite a pair-matching process that contributed to attenuating differences in baseline characteristics and clinical variables, a complete elimination of residual confounding is difficult to achieve in observational studies. In addition, patients who have been moved from one hospital to another would appear twice and could not be detected. Finally, in the study population we included only patients aged 35 or over because the prevalence of T2DM in Spain under this age is very low [56]. In our database we found that in Spain from 2016 to 2018 a total of 1103 individuals aged 18 to 34 years with a primary diagnosis of ischemic stroke were hospitalized. Among these patients, 45 had a code for diabetes, with 41 corresponding to T1DM and only four to T2DM, therefore confirming that the influence of T2DM on ischemic stroke among young adults in our country is very small.

## 5. Conclusions

In summary, we have observed that both men and women who suffer from T2DM have a higher incidence of hospitalizations for ischemic stroke than non-diabetic subjects. As among subjects without diabetes, hospitalizations are more frequent in diabetic men than in women with this disease, although it is T2DM women those who suffer the highest in-hospital mortality. We believe that future research should deepen the analysis of the possible differences in the treatments and care received between diabetic men and diabetic women after ischemic stroke and efforts should focus on eliminating these sex-related disparities in our health system.

## Figures and Tables

**Table 1:** Incidence of ischemic stroke according to presence of type 2 diabetes mellitus (T2DM), sex, and age groups.

		No T2DM	T2DM	
Sex	Age Group	*n* (Inc/10^5^)	*n* (Inc/10^5^)	*p*-Value
Male	35–49 years	4116 (4.28)	646 (28.46)	<0.001
	50–64 years	15,197 (23.97)	6222 (74.85)	<0.001
	65–79 years	23,716 (65.69)	13,153 (139.65)	<0.001
	≥80 years	20,854 (181.1)	8620 (290.15)	<0.001
	All age groups	63,883 (30.83)	28,641 (124.68)	<0.001
Female	35–49 years	2289 (2.41)	222 (11.44)	<0.001
	50–64 years	6042 (8.83)	1786 (32.26)	<0.001
	65–79 years	16,365 (35.97)	7763 (74.87)	<0.001
	≥80 years	32,807 (175.5)	12,457 (261.57)	<0.001
	All age groups	57,503 (25.29)	22,228 (98.33)	<0.001
Total	35–49 years	6405 (3.35)	868 (20.62)	<0.001
	50–64 years	21,239 (16.11)	8008 (57.83)	<0.001
	65–79 years	40,081 (49.12)	20,916 (105.71)	<0.001
	≥80 years	53,661 (177.63)	21,077 (272.55)	<0.001
	All age groups	121,386 (27.93)	50,869 (111.61)	<0.001

T2DM, type 2 diabetes mellitus; Inc/105, incidence per 100,000 people with or without T2DM. *p* values for comparison of the incidence between patients with and without T2DM using Poisson Regression adjusted by age and sex when required.

**Table 2:** Clinical characteristics, use of therapeutic procedures, and hospital outcomes before and after matching by age and ischemic stroke type (ICD 10) for men patients with ischemic stroke.

Variables	Before Matching			After Matching		
	No T2DM	T2DM	*p*-Value	No T2DM	T2DM	*p*-Value
IS by thrombosis of precerebral arteries n (%)	2111(3.3)	1048(3.66)	0.233	993(3.5)	993(3.5)	NA
IS by embolism of precerebral arteries n (%)	1237(1.94)	446(1.56)	0.326	419(1.48)	419(1.48)	NA
IS by unspecified occlusion or stenosis of precerebral arteries n (%)	4027(6.3)	2022(7.06)	0.015	1991(7.01)	1991(7.01)	NA
IS by thrombosis of cerebral arteries n (%)	8652(13.54)	4266(14.89)	<0.001	4191(14.76)	4191(14.76)	NA
IS by embolism of cerebral arteries n (%)	12,532(19.62)	4489(15.67)	<0.001	4470(15.74)	4470(15.74)	NA
IS by unspecified occlusion or stenosis of cerebral arteries n (%)	22,196(34.74)	9976(34.83)	0.706	9958(35.06)	9958(35.06)	NA
IS by cerebral venous thrombosis, non-pyogenic n (%)	53(0.08)	27(0.09)	0.988	12(0.04)	12(0.04)	NA
Other cerebral infarction n (%)	2788(4.36)	1263(4.41)	0.044	1263(4.45)	1263(4.45)	NA
Cerebral infarction, unspecified n (%)	10,287(16.1)	5104(17.82)	<0.001	5104(17.97)	5104(17.97)	NA
Age, mean (SD)	71.37(13.22)	72.48(10.92)	<0.001	72.51(10.92)	72.51(10.92)	NA
CCI, mean (SD)	0.7(0.66)	0.84(0.74)	<0.001	0.71(0.67)	0.84(0.74)	<0.001
Obesity, n (%)	3225(5.05)	2640(9.22)	<0.001	1367(4.81)	2614(9.2)	0.405
Hypertension, n (%)	32,279(50.53)	18,328(63.99)	<0.001	14,780(52.04)	18,179(64.01)	<0.001
Lipid metabolism disorders, n (%)	22,392(35.05)	15,285(53.37)	<0.001	10,310(36.3)	15,164(53.39)	<0.001
Renal disease, n (%)	4791(7.5)	3626(12.66)	<0.001	2200(7.75)	3603(12.69)	<0.001
Atrial fibrillation, n (%)	15,257(23.88)	6012(20.99)	<0.001	6342(22.33)	5980(21.06)	<0.001
Congestive heart failure, n (%)	3503(5.48)	1990(6.95)	<0.001	1491(5.25)	1972(6.94)	<0.001
Peripheral vascular disease, n (%)	3774(5.91)	2564(8.95)	<0.001	1796(6.32)	2546(8.96)	<0.001
Acute myocardial infarction, n (%)	2818(4.41)	1967(6.87)	<0.001	1309(4.61)	1949(6.86)	<0.001
Dementia, n (%)	1969(3.08)	1054(3.68)	<0.001	898(3.16)	1049(3.69)	<0.001
Anemia, n (%)	1469(2.3)	900(3.14)	<0.001	662(2.33)	893(3.14)	<0.001
Alcohol abuse, n (%)	7325(11.47)	3017(10.53)	<0.001	3362(11.84)	2983(10.5)	0.103
Depression, n (%)	1960(3.07)	1005(3.51)	<0.001	866(3.05)	999(3.52)	<0.001
Mechanical ventilation, n (%)	1857(2.91)	637(2.22)	<0.001	858(3.02)	628(2.21)	<0.001
Endovascular thrombectomy, n (%)	3142(4.92)	834(2.91)	<0.001	1268(4.46)	827(2.91)	0.034
Thrombolytic therapy, n (%)	5017(7.85)	1559(5.44)	<0.001	2070(7.29)	1547(5.45)	<0.001
LOHS, median (IQR)	7(7)	7(7)	0.474	6(7)	7(7)	0.279
In-hospital mortality, n (%)	5671(8.88)	2358(8.23)	0.001	2473(8.71)	2336(8.23)	<0.001

IS, ischemic stroke; T2DM, type 2 diabetes mellitus; CCI, Charlson comorbidity index; LOHS, length of hospital stay.

**Table 3:** Clinical characteristics, use of therapeutic procedures, and hospital outcomes before and after matching by age and ischemic stroke type (ICD 10) for women patients with ischemic stroke

Variables	Before Matching			After Matching		
	No T2DM	T2DM	*p*-Value	No T2DM	T2DM	*p*-Value
IS by thrombosis of precerebral arteries n (%)	1103(1.92)	507(2.28)	0.284	459(2.09)	459(2.09)	NA
IS by embolism of precerebral arteries n (%)	1074(1.87)	367(1.65)	0.557	356(1.62)	356(1.62)	NA
IS by unspecified occlusion or stenosis of precerebral arteries n (%)	2408(4.19)	946(4.26)	0.775	922(4.19)	922(4.19)	NA
IS by thrombosis of cerebral arteries n (%)	6370(11.08)	2902(13.06)	<0.001	2807(12.76)	2807(12.76)	NA
IS by embolism of cerebral arteries n (%)	14,638(25.46)	4815(21.66)	<0.001	4795(21.79)	4795(21.79)	NA
IS by unspecified occlusion or stenosis of cerebral arteries n (%)	20,879(36.31)	8032(36.13)	0.478	8015(36.43)	8015(36.43)	NA
IS by cerebral venous thrombosis, non-pyogenic n (%)	73(0.13)	19(0.09)	0.878	8(0.04)	8(0.04)	NA
Other cerebral infarction n (%)	1965(3.42)	798(3.59)	0.572	798(3.63)	798(3.63)	NA
Cerebral infarction, unspecified n (%)	8993(15.64)	3842(17.28)	<0.001	3842(17.46)	3842(17.46)	NA
Age, mean (SD)	78.29(12.69)	79.08(9.98)	<0.001	79.17(9.91)	79.17(9.91)	NA
CCI, mean (SD)	0.66(0.51)	0.79(0.68)	<0.001	0.65(0.5)	0.79(0.68)	<0.001
Obesity, n (%)	3650(6.35)	2732(12.29)	<0.001	1395(6.34)	2683(12.19)	<0.001
Hypertension, n (%)	31,227(54.3)	14,911(67.08)	<0.001	12,348(56.12)	14,765(67.11)	<0.001
Lipid metabolism disorders, n (%)	20,169(35.07)	11,706(52.66)	<0.001	8072(36.69)	11,570(52.59)	<0.001
Renal disease, n (%)	4840(8.42)	3144(14.14)	<0.001	1846(8.39)	3121(14.19)	<0.001
Atrial fibrillation, n (%)	20,300(35.3)	7352(33.08)	<0.001	7503(34.1)	7312(33.23)	0.054
Congestive heart failure, n (%)	3915(6.81)	2008(9.03)	<0.001	1417(6.44)	1996(9.07)	<0.001
Peripheral vascular disease, n (%)	1491(2.59)	804(3.62)	<0.001	566(2.57)	792(3.6)	<0.001
Acute myocardial infarction, n (%)	1242(2.16)	888(3.99)	<0.001	471(2.14)	881(4)	<0.001
Dementia, n (%)	4153(7.22)	1926(8.66)	<0.001	1620(7.36)	1914(8.7)	<0.001
Anemia, n (%)	2324(4.04)	1344(6.05)	<0.001	846(3.85)	1332(6.05)	<0.001
Alcohol abuse, n (%)	740(1.29)	182(0.82)	<0.001	259(1.18)	178(0.81)	<0.001
Depression, n (%)	4512(7.85)	1792(8.06)	0.312	1829(8.31)	1771(8.05)	0.313
Mechanical ventilation, n (%)	1167(2.03)	398(1.79)	0.029	466(2.12)	392(1.78)	0.011
Endovascular thrombectomy, n (%)	2958(5.14)	644(2.9)	<0.001	1104(5.02)	641(2.91)	<0.001
Thrombolytic therapy, n (%)	4404(7.66)	1261(5.67)	0.000	1618(7.35)	1247(5.67)	0.000
LOHS, median (IQR)	7(7)	7(8)	0.033	7(7)	7(8)	0.002
In-hospital mortality, n (%)	7801(13.57)	2936(13.21)	0.185	2840(12.91)	2922(13.28)	0.247

IS, ischemic stroke; T2DM, type 2 diabetes mellitus; CCI, Charlson comorbidity index; LOHS, length of hospital stay.

**Table 4:** Clinical characteristics, use of therapeutic procedures, and hospital outcomes before and after matching by age (ICD 10) after ischemic stroke among patients with T2DM according to sex.

Variables	Before Matching			After Matching		
	T2DM Men	T2DM Women	*p*-Value	T2DM Men	T2DM Women	*p*-Value
35–49 years, n (%)	646(2.26)	222(1)	0.001	189(1.08)	189(1.08)	NA
50–64 years, n (%)	6222(21.72)	1786(8.03)	<0.001	1749(9.98)	1749(9.98)	NA
65–79 years, n (%)	13,153(45.92)	7763(34.92)	<0.001	7515(42.87)	7515(42.87)	NA
≥80 years, n (%)	8620(30.1)	12,457(56.04)	<0.001	8076(46.07)	8076(46.07)	NA
Age, mean (SD)	72.48(10.92)	79.08(9.98)	<0.001	76.95(9.60)	76.95(9.60)	NA
CCI, mean (SD)	0.84(0.74)	0.79(0.68)	<0.001	0.76(0.67)	0.83(0.72)	<0.001
Obesity, n (%)	2640(9.22)	2732(12.29)	<0.001	1278(7.29)	2370(13.52)	<0.001
Hypertension, n (%)	18,328(63.99)	14,911(67.08)	<0.001	11,040(62.98)	11,922(68.01)	<0.001
Lipid metabolism disorders, n (%)	15,285(53.37)	11,706(52.66)	0.114	9035(51.54)	9517(54.29)	<0.001
Renal disease, n (%)	3626(12.66)	3144(14.14)	<0.001	2587(14.76)	2288(13.05)	<0.001
Atrial fibrillation, n (%)	6012(20.99)	7352(33.08)	<0.001	4556(25.99)	5173(29.51)	<0.001
Congestive heart failure, n (%)	1990(6.95)	2008(9.03)	<0.001	1389(7.92)	1461(8.33)	0.159
Peripheral vascular disease, n (%)	2564(8.95)	804(3.62)	<0.001	1553(8.86)	671(3.83)	<0.001
Acute myocardial infarction, n (%)	1967(6.87)	888(3.99)	<0.001	1246(7.11)	699(3.99)	<0.001
Dementia, n (%)	1054(3.68)	1926(8.66)	<0.001	874(4.99)	1331(7.59)	<0.001
Anemia, n (%)	900(3.14)	1344(6.05)	<0.001	653(3.73)	980(5.59)	<0.001
Alcohol abuse, n (%)	3017(10.53)	182(0.82)	<0.001	1357(7.74)	167(0.95)	<0.001
Depression, n (%)	1005(3.51)	1792(8.06)	<0.001	577(3.29)	1516(8.65)	<0.001
Mechanical ventilation, n (%)	637(2.22)	398(1.79)	0.001	343(1.96)	353(2.01)	0.702
Endovascular thrombectomy, n (%)	834(2.91)	644(2.9)	0.922	476(2.72)	526(3)	0.109
Thrombolytic therapy, n (%)	1559(5.44)	1261(5.67)	0.261	969(5.53)	1033(5.89)	0.141
LOHS, median (IQR)	7(7)	7(7)	0.924	7(7)	7(7)	0.183
In-hospital mortality, n (%)	2358(8.23)	2963(13.21)	<0.001	1788(10.20)	1955(11.15)	0.004

T2DM: Type 2 diabetes mellitus. CCI: Charlson comorbidity index; LOHS: length of hospital stay.

**Table 5:** Multivariable logistic regression analysis of factors associated with in-hospital mortality among patients with T2DM according to sex.

	Male	Female	Both
35–49 years	1	1	1
50–64 years	1.78(1–3.17)	1.14(0.5–2.6)	0.96(0.53–1.75)
65–79 years	3.3(1.88–5.79)	2.13(0.97–4.68)	1.87(1.05–3.33)
≥80 years	7.49(4.26–13.17)	5.79(2.64–12.69)	4.3(2.42–7.63)
Obesity	0.8(0.67–0.96)	0.85(0.74–0.98)	0.83(0.73–0.95)
Renal disease	1.34(1.17–1.52)	1.17(1.03–1.32)	1.24(1.12–1.38)
Atrial fibrillation	1.5(1.36–1.66)	1.61(1.48–1.75)	1.48(1.37–1.59)
Congestive heart failure	2.09(1.83–2.4)	1.83(1.62–2.06)	2.03(1.82–2.25)
Peripheral vascular disease	1.07(0.92–1.24)	1.08(0.87–1.35)	1.07(0.92–1.24)
Acute myocardial infarction	1.38(1.18–1.61)	1.38(1.15–1.67)	1.32(1.15–1.52)
Dementia	1.76(1.48–2.1)	1.69(1.49–1.9)	1.73(1.53–1.94)
Mechanical ventilation	14.97(12.41–18.07)	11.68(9.26–14.71)	11.6(9.74–13.81)
Thrombolytic therapy	0.68(0.55–0.85)	0.63(0.51–0.78)	0.68(0.57–0.81)
Female sex	NA	NA	1.12(1.04–1.21)

T2DM, type 2 diabetes mellitus. Only variables with significant results in the multivariable regression are shown in the table. NA, not available.

## Data Availability

According to the contract signed with the Spanish Ministry of Health and Social Services, which provided access to the databases from the Spanish National Hospital Database (Conjunto Mínimo Basico de Datos; CMBD), we cannot share the databases with any other investigator, and we have to destroy the databases once the investigation has concluded. Consequently, we cannot upload the databases to any public repository. However, any investigator can apply for access to the databases by filling out the questionnaire available at http://www.msssi.gob.es/estadEstudios/estadisticas/estadisticas/estMinisterio/SolicitudCMBDdocs/Formulario_Peticion_Datos_CMBD.pdf. All other relevant data are included in the paper.

## Supplementary Materials

The following are available online at https://www.mdpi.com/article/10.3390/ijerph18073659/s1, Table S1: International Classification of Disease 10th edition (ICD-10) codes for the clinical diagnosis and procedures used in this investigation, Table S2: Logistic regression factors associated with IHM after myocardial infarction among all patients and according to the presence of T2DM to assess the sex differences.
