# Supplementary material for: Sex-Related Disparities in the Incidence and Outcomes of Ischemic Stroke among Type 2 Diabetes Patients. A Matched-Pair Analysis Using the Spanish National Hospital Discharge Database for Years 2016–2018
Source: Int J Environ Res Public Health. 2021 Apr 1;18(7):3659. doi: 10.3390/ijerph18073659 (PMC8037293; doi:10.3390/ijerph18073659)
Supplement: Supplementary file 1 [file ijerph-18-03659-s001.pdf]

Table S1. International Classification of Disease 10th edition (ICD-10) codes for the clinical diagnosis and procedures used in this investigation.

**Clinical diagnosis and procedures**	**ICD-10 codes**
IS by thrombosis of precerebral arteries	I63.0; I63.00; I63.01; I63.01X; I63.02; I63.03; I63.03X; I63.09
IS by embolism of precerebral arteries	I63.1; I63.10; I63.11; I63.11X; I63.12; I63.13; I63.13X; I63.19
IS by unspecified occlusion or stenosis of precerebral arteries	I63.2; I63.20; I63.21; I63.21X; I63.22; I63.23; I63.23X; I63.29
IS by thrombosis of cerebral arteries	I63.3; I63.30; I63.31; I63.31X; I63.32; I63.32X; I63.33; I63.33X; I63.34; I63.34X; I63.39
IS by embolism of cerebral arteries	I63.4; I63.40; I63.41; I63.41X; I63.42; I63.42X; I63.43; I63.43X; I63.44; I63.44X; I63.49
IS by unspecified occlusion or stenosis of cerebral arteries	I63.5; I63.50; I63.51; I63.51X; I63.52; I63.52X; I63.53; I63.53X; I63.54; I63.54X; I63.59
IS by cerebral venous thrombosis, non pyogenic	I63.6
Other cerebral infarction	I63.8; I63.81; I63.89
Cerebral infarction, unspecified	I63.9
Obesity	E66.X
Hypertension	I10, I16.6
Lipid metabolism disorders	E78.0X-E78.5
Atrial fibrillation	I48.0, I48.1, I48.2, I48.91
Anemia	D50.0, D50.8, D50.9, D51.x-D53.x
Alcohol abuse	F10, E52, G62.1, I42.6, K29.2, K70.0, K70.3, K70.9, T51.x, Z50.2, Z71.4, Z72.1
Depression	F20.4, F31.3-F31.5, F32.x, F33.x, F34.1, F41.2, F43.2
Mechanical ventilation	5A1945Z, 5A1955Z, 5A1935Z,5A09357, 5A09457, 5A09557
Endovascular thrombectomy	03CG3ZZ, 03CH3ZZ, 03CJ3ZZ, 03CK3ZZ, 03CL3ZZ, 03CM3ZZ, 03CN3ZZ, 03CP3ZZ, 03CQ3ZZ
Thrombolytic therapy	3E03317, 3E04317, 3E05317, 3E06317; Z9282

IS: ischemic stroke.

Table S2. Logistic regression factors associated with IHM after myocardial infarction among all patients and according to the presence of T2DM to assess the sex differences.

	**Male**	**Female**
35–49 years	1	1
50–64 years	1.8(1.22–2.65)	1.22(0.69–2.18)
65–79 years	3.15(2.16–4.61)	2.15(1.23–3.74)
≥80 years	7.45(5.1–10.89)	5.89(3.38–10.26)
Obesity	0.81(0.7–0.94)	0.84(0.75–0.95)
Renal disease	1.21(1.1–1.33)	1.23(1.12–1.34)
Atrial fibrillation	1.37(1.28–1.47)	1.54(1.45–1.64)
Congestive heart failure	1.95(1.76–2.15)	1.76(1.61–1.93)
Acute myocardial infarction	1.39(1.24–1.57)	1.4(1.21–1.63)
Dementia	1.88(1.66–2.14)	1.65(1.51–1.8)
Mechanical ventilation	15.58(13.76–17.65)	13.47(11.48–15.79)
Thrombolytic therapy	0.77(0.67–0.88)	0.62(0.53–0.71)
T2DM	1.03(0.97–1.1)	1.06(0.99–1.13)

T2DM: Type 2 diabetes mellitus.
